# Size-Specific Dose Estimates of Radiation Based on Body Weight and Body Mass Index for Chest and Abdomen-Pelvic CTs

**DOI:** 10.1155/2020/6046501

**Published:** 2020-07-10

**Authors:** Jian Xu, Xiangquan Wang, Panfeng Yang, Kuangnan Luo, Xiaolong He

**Affiliations:** ^1^Department of Radiology, Zhejiang Provincial People's Hospital, People's Hospital of Hangzhou Medical College, Hangzhou, Zhejiang, China; ^2^Shenshi Technology, Co., Ltd, Hangzhou, Zhejiang, China; ^3^Department of Radiology, Quzhou People's Hospital, Quzhou, Zhejiang, China

## Abstract

**Background:**

To correlate body weight, body mass index (BMI), and water-equivalent diameter (*d*_w_) and to assess size-specific dose estimates (SSDEs) based on body weight and BMI for chest and abdomen-pelvic CT examinations.

**Methods:**

An in-house program was used to calculate *d*_w_, size-dependent conversion factor (*f*), and SSDE for 1178 consecutive patients undergoing chest and abdomen-pelvic CT examinations. Associations among body weight, BMI, and *d*_w_ were determined, and linear equations were generated using linear regression analysis of the first 50% of the patient population. SSDEs (SSDE_weight_ and SSDE_BMI_) were calculated based on body weight and BMI as *d*_w_ surrogates on the second 50% of the patient population. Mean root-mean-square errors of SSDE_weight_ and SSDE_BMI_ were computed with SSDE from the axial images as reference values.

**Results:**

Both body weight and BMI correlated strongly with *d*_w_ for the chest (*r* = 0.85, 0.87, all *p* < 0.001) and abdomen-pelvis (*r* = 0.85, 0.86, all *p* < 0.001). Mean values of SSDE_weight_ and SSDE_BMI_ based on the linear equations for body weight, BMI, and *d*_w_ were in close agreement with SSDE from the axial images, with overall mean root-mean-square errors of 0.62 mGy (6.10%) and 0.57 mGy (5.65%), for chest, and 0.76 mGy (5.61%) and 0.71 mGy (5.22%), for abdomen-pelvis, respectively.

**Conclusions:**

Both body weight and BMI, serving as *d*_w_ surrogates, can be used to calculate SSDEs in the chest and abdomen-pelvis CT examinations, providing values comparable to SSDEs from the axial images, with an overall mean root-mean-square error of less than 0.76 mGy or 6.10%.

## 1. Introduction

The increased risk of radiation-induced cancer from CT scans is a major concern for the medical community [1–4]. Compared with other radiologic imaging modalities, CT scanning is associated with a higher radiation dosage because of the scanner type, operation condition, scan protocol, and diagnostic reliability [2, 5]. CT examinations account for up to 60% of the total medical radiation dose; however, CT examinations only contribute to approximately 6% of radiological procedures [6, 7]. Thus, it is necessary to execute the scan protocol in agreement with the ALARA principle. Unfortunately, the implementation of the ALARA principle is challenged because the dose metrics routinely used in the clinical setting, such as volume CT dose index (CTDI_vol_) and dose length product (DLP), cannot provide the exact radiation dose absorbed by the patient [8, 9]. CTDI_vol_ is susceptive to scanning parameters (e.g., kVp, mAs, pitch, and filter) and is a standardized dose metric derived from a cylindrical acrylic polymethyl methacrylate phantom with a diameter of 16 or 32 cm, whereas DLP is derived by multiplying CTDI_vol_ by the scan length. Owing to the fact that the parameter does not account for factors related to patients, CTDI_vol_ only reflects the average output level of the CT scanner for certain scan settings and is appropriately adapted to its purposes for the comparison of different CT scanners and scanning protocols.

The concept of size-specific dose estimate (SSDE) was introduced by the American Association of Physicists in Medicine (AAPM) to refine CTDI_vol_ on the basis of patient body size [10, 11]. In the radiation dose structured report, the CTDI_vol_ is normalized to SSDE using size-dependent conversion factor (*f*) based on patient size expressed through an effective diameter (*d*_eff_) or water-equivalent diameter (*d*_w_). Measured in centimeters, *d*_eff_ can be computed in combination with the anterior-posterior (AP) and lateral (LAT) dimensions. Measurements of AP and LAT are performed using the axial images or CT scanogram, as recommended by AAPM [10], whereas *d*_w_ requires delineating the region of interest and measuring CT attenuation on the axial images slice by slice to accurately calculate SSDE [11], which is time-consuming and tedious. Recently, a series of studies focused on simplifying the calculation of SSDE via correlations between *d*_eff_ and weight or body mass index (BMI) [12–16]. Because of the limitation of *d*_eff_, which only represents the geometric size of the patient, the accuracy of SSDE is inferior to that calculated using *d*_w_. Consequently, a number of efforts [17–19] have been made to investigate the relationship between *d*_eff_ and *d*_w_ with the aims of using *d*_eff_ as the *d*_w_ surrogate and achieving an accurate SSDE, comparable to that derived from the *d*_w_ of the axial images. However, the methodologies still required measuring *d*_eff_ on the axial images, and a prescan SSDE could not be obtained. In addition, until recently, CT scanner manufacturers have not included SSDE on the CT scanner console display. Therefore, optimization of CT protocols on the basis of SSDE is impractical, and radiologists cannot use SSDE to assess the risk versus benefits of a patient undergoing a CT examination prior to imaging.

Sarmento et al.'s study reported that *d*_w_ is a function of patient weight, suggesting that the calculation of SSDEs could be simplified using weight [20, 21]. To the best of our knowledge, there have been no previous studies on the calculation of SSDE from the chest and abdomen-pelvic CT examinations based on the correlations of body weight, BMI, and *d*_w_. Hence, this study is aimed at determining the correlations between two biometric indicators of weight, BMI with *d*_w_ and verifying the accuracy of an SSDE predicted using weight and BMI as surrogate body size metrics for *d*_w_ for the chest and abdomen-pelvic CT examinations of adult patients.

## 2. Materials and Methods

### 2.1. Patient Population

This retrospective study was executed with approval from Zhejiang Provincial People's Hospital Ethics Committee. The requirement of written informed consent was waived in accordance with hospital policies for clinical retrospective studies.

A Picture Archive and Communication System (PACS, Greenlander version 6.0, Mindray Healthcare, Shenzhen, China) terminal was used to extract axial images and radiation dose structured reports. For the period between January and August 2019, the records of consecutive inpatients undergoing chest and abdomen-pelvic CT examinations were electronically retrieved. Patients with nondiagnostic images, truncated images, severe motion artifacts, and in-body or in-skin metal objects were excluded from this study. Patients with renal insufficiency (serum creatinine > 1.5 mg/dL) or known allergic reactions to iodinated contrast medium for contrast-enhanced CT were excluded from the study. The final study cohort was composed of a total of 1178 patients, including those that underwent chest CT (*n* = 616) and abdomen-pelvic CT (*n* = 562). Among the patients, there were 782 males and 396 females with a mean age of 58.50 ± 13.20 years (range, 18.00-90.00), mean weight of 63.07 ± 11.73 kg (range, 35.00-110.00), and mean BMI of 23.00 ± 3.41 kg/m^2^ (range, 14.10-36.84). The first 50% of patients in chronological order, 308 and 281 individuals that received chest and abdomen-pelvic CT examinations, respectively, served as a model sample to generate regression equations of body weight, BMI, and *d*_w_. The second 50% of patients, referred to as the verified sample, were used to confirm the feasibility of calculating SSDE based on body weight and BMI.

In accordance with our hospital's clinical practice, the weight and height of inpatients had been documented in the electronic medical records and displayed in the electronic application form for CT examination. BMI was calculated as weight divided by height squared (kg/m^2^). The body habitus was divided into four types according to the redefined World Health Organization (WHO) criteria for the Asia-Pacific Region [22]: B1: underweight, BMI < 18.49 (*n* = 102); B2: normal weight, 18.5 < BMI < 22.9 (*n* = 492); B3: overweight, 23.0 < BMI < 29.9 (*n* = 559); and B4: obese, BMI > 30 (*n* = 25).

### 2.2. CT Acquisition

CT examinations were performed using a 40-slice scanner and a 128-slice scanner (Definition AS and Definition AS+, Siemens AG, Healthcare Sector, Forchheim, Germany). The scan parameters of both chest and abdomen-pelvic CT examinations were 100 or 120 kVp tube voltage, 0.5-second rotation-time, and a pitch factor of 1.35. Automatic tube current modulation (ATCM) was activated to reduce the radiation dose for all scans. ATCM is a combination of applications that reduce dose exposure in four dimensions (CARE Dose 4D), created by the manufacturer Siemens. Axial images were reconstructed with a standard tissue algorithm (B30f), a 512 × 512 matrix, 400 × 400 mm FOV, and 5/5 mm slice thickness and increment. The scan range for the chest CT was from the lung apices to the costophrenic angle, and the scan range for abdomen-pelvis CT included diaphragmatic top and pubic symphysis. The aforementioned scan parameters were set according to standard department CT protocols for adults.

### 2.3. Calculation of Size-Specific Dose Estimates

An in-house program based on MATLAB (MathWorks, Natick, Mass) was developed to automatically calculate *d*_w_, *f*, and SSDE slice by slice and provide mean values across all axial images in accordance with the methodologies detailed in the AAPM report 220 [11]. The automatic calculation process included several steps. The first step was a threshold-based segmentation with a threshold of -500 HU, and axial images were converted from grayscale to binary using an image threshold. The second step was removal of the patient table from the binary image using the largest connected area method. The third step was to label the entire region of interest with the hole-filled technique. The last step was to delineate the object boundary and calculate *d*_w_, *f*, and SSDE, including a single value slice by slice and mean values across all slices. The automatic calculation of *d*_w_, *f*, and SSDE required the following formulas [11]:

(1) $$\begin{array}{c} \text{CTD}{\text{I}}_{\text{vol}\left(\text{s}\right)}=\frac{\text{mAs}\left(\text{s}\right)}{\text{mAs}\left(\text{a}\right)}\cdot {\text{CTDI}}_{\text{vol}}, \end{array}$$

(2) $$\begin{array}{c} d_{\text{w}}=2\cdot \sqrt{\left(\frac{\text{C}{\text{T}}_{\text{ROI}}}{1000}+1\right)\cdot \frac{A_{\text{ROI}}}{\pi }}, \end{array}$$

(3) $$\begin{array}{c} f=4.378094\times \exp\left(-0.04331124\times d_{\text{w}}\right), \end{array}$$

(4) $$\begin{array}{c} \text{SSDE}=f\times {\text{CTDI}}_{\text{vol}\left(\text{s}\right)}, \end{array}$$

where CTDI_vol(s)_ is the slice CTDI_vol_, mAs(s) is the actual mAs per slice, mAs(a) is the averaged mAs displayed in the radiation dose structured report, CTDI_vol_ is the volume CT dose index displayed in the radiation dose structured report, *d*_w_ is the water-equivalent diameter, CT_ROI_ is the CT number of the whole axial image, *A*_ROI_ is the area of the axial image, *f* is the size-dependent conversion factor, and SSDE is the size-specific dose estimate.

To analyze the radiation dose, we listed three SSDE measurements as follows: SSDE, SSDE_weight_, and SSDE_BMI_. SSDE_weight_ and SSDE_BMI_, defined as the new radiation dose indicators, required three steps. Firstly, linear regression analysis was used to generate linear equations (*y* = *a* + *bx*) for body weight and BMI with *d*_w_ across all modeled patients. Secondly, new body size metrics of *d*_w_, defined as *d*_w,weight_ and *d*_w,BMI_, were predicted from body weight or BMI, after which the corresponding *f* was obtained using formula (2). Finally, SSDE_weight_ and SSDE_BMI_ were computed via *f* multiplied by CTDI_vol(s)_. All three steps were performed with a Microsoft Excel 2016 spreadsheet embedded with all linear equations of *d*_w_ and two biometric indicators of body weight and BMI. Only plain axial CT images were analyzed in this study.

### 2.4. Statistical Analysis

**Figure 1:**
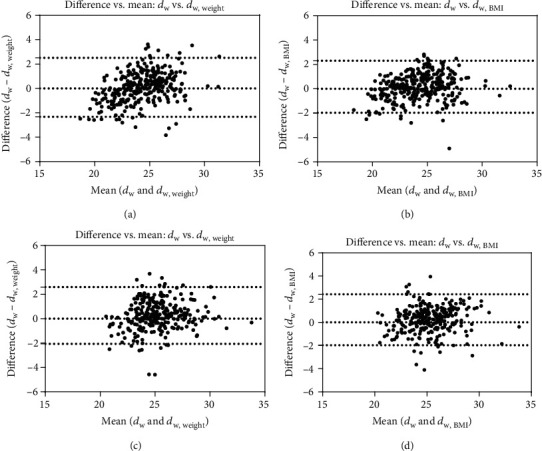
Bland-Altman plots for the measured *d*_w_ and the predicted *d*_w_. (a, b) Plots for chest CT; (c, d) plots for abdomen-pelvis CTs.

Statistical analyses were performed using the statistical software SPSS 22.0 (IBM Corp., Armonk, NY, USA). The normality of continuous variables was determined using the Shapiro-Wilk test. Continuous variables with normal distributions were described as the mean ± standard deviation, and dichotomous variables were reported as counts and percent. The Spearman Rho test was used to assess the correlation of variables that do not follow normal distribution and Pearson's test for those which follow normal distribution. Associations between age and *d*_w_, body weight, and BMI with *d*_w_ were assessed with Spearman's rank correlation analysis and Pearson's correlation analysis, respectively. Steiger's test was used to compare the correlation coefficients of body weight and BMI with *d*_w_ as the common variable in chest and abdomen-pelvis. A paired *t*-test was used for SSDE_weight_ and SSDE_BMI_ in chest and abdomen-pelvic CT examinations. The Bland-Altman test was performed to determine the accuracy of the linear equations compared with the measured values (Figure 1). With SSDE from the axial images as reference values, the mean root-mean-square errors of SSDE_weight_ and SSDE_BMI_ were calculated for all verified patients. A *p* value of less than 0.05 was considered to indicate a statistically significant difference.

## 3. Results

### 3.1. Patient Characteristics, Body Size, and Radiation Dose

The number of CT examinations for chest and abdomen-pelvis was 264 and 239 on the 40-slice scanner and 352 and 323 on the 128-slice scanner, respectively. Patient demographics are listed in Table 1. CTDI_vol_ and values of *d*_w_, *f*, and SSDE, calculated using the in-house program, are shown in Table 2. Measurements of *d*_w_ for both chest and abdomen-pelvis were considerably less than the 32 cm diameter of the standard AAPM phantom used to determine CTDI_vol_.

### 3.2. Correlation of *d*_w_ with Two Biometric Characteristics

**Figure 2:**
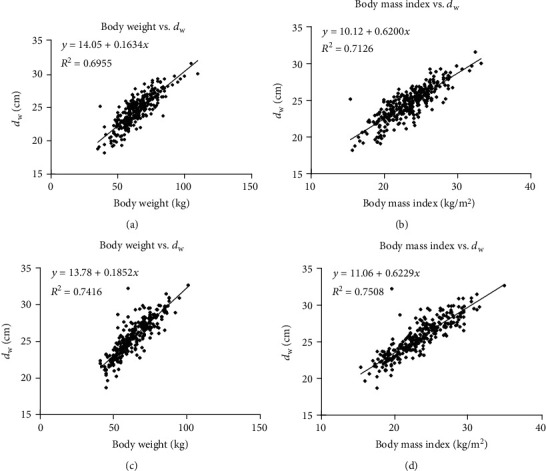
Scatterplots representing correlations between *d*_w_ and two body size metrics of body weight and BMI. (a) Correlation of body weight with *d*_w_ for chest. (b) Correlation of BMI with *d*_w_ for chest. (c) Correlation of body weight with *d*_w_ for abdomen-pelvis. (d) Correlation of BMI with *d*_w_ for abdomen-pelvis. Linear equations and coefficients of determination (*R*^2^) from the linear regression analysis are also shown.

Spearman's correlation analysis showed that no statistically significant correlations occurred between age and *d*_w_ for chest and abdomen-pelvis CT (*r* = 0.014, -0.010, all *p* > 0.05). Pearson's correlation analysis indicated that both body weight and BMI were strongly correlated with *d*_w_ for the chest (*r* = 0.85, 0.87, all *p* < 0.001) and abdomen-pelvis (*r* = 0.85, 0.86, all *p* < 0.001). Steiger's test showed a statistically significant difference associated with the correlation coefficients of body weight and BMI with *d*_w_ as a common variable for the chest (*z* = −1.154, *p* < 0.05). However, no significant difference was observed for two correlation coefficients of body weight and BMI and *d*_w_ as a common variable for abdomen-pelvis using Steiger's test (*z* = −0.711, *p* > 0.05).  Figure 2 shows the correlations and linear regressions relating to body weight, BMI, and *d*_w_ automatically derived from axial images of modeled patients. The Bland-Altman test indicated that there was good agreement between measured *d*_w_ and predicted values based on the linear equations for chest and abdomen-pelvis CTs (Figure 1).

### 3.3. Accuracy of SSDE_weight_ and SSDE_BMI_

SSDE_weight_ and SSDE_BMI_ were 10.41 ± 3.06 mGy and 10.42 ± 2.98 mGy in chest CT examination, respectively (*t* = −0.413, *p* > 0.05); SSDE_weight_ and SSDE_BMI_ were 13.81 ± 1.98 mGy and 13.79 ± 1.95 mGy in abdomen-pelvic CT examination, respectively (*t* = 0.633, *p* > 0.05). Compared with reference values of SSDE, slightly greater dispersions were observed for SSDE_weight_ and SSDE_BMI_ with respect to standard deviation, range, and coefficient of variation, as shown in Table 3. Maximal mean root-mean-square errors of SSDE_weight_ and SSDE_BMI_ were less than 11%, as shown in Table 4.

## 4. Discussion

Prior to estimating patients' absorbed radiation dose, accurate measurements of patient size are necessary, as the radiation dose is closely related to patient factors as well as the output of the CT scanner. Similarly to previous studies [18, 23], we found that the actual patient sizes for chest and abdomen-pelvis were considerably less than the 32 cm diameter standard AAPM phantom. Irrespective of inhomogeneous X-ray attenuation, a 32 cm diameter cannot accurately represent a realistic patient size in terms of geometric dimension. Hence, the radiation doses expressed via CTDI_vol_ were underestimated compared to actual values [18, 23, 24].

Previous studies have shown that the measurement of some patient size metrics, including age, height, body weight, BMI, body circumference, and body diameter, could be integrated into routine clinical practice for dose optimization [15, 26, 27]. As suggested by AAPM, *d*_eff_ and *d*_w_ are suitable for quantifying patient size [10, 11]. Compared with *d*_eff_, *d*_w_ can be considered the gold standard for patient size, as it can be adapted to different body shapes, including complex deformities and asymmetric contours, and accounts for X-ray attenuations of heterogeneous tissues within the body. However, owing to the unavailability of commercial or in-house software in many health institutions worldwide, the measurement of *d*_w_ is often a complex and time-consuming process for routine CT radiation dose calculations in radiological practice. The need for a simplified and efficient method to obtain *d*_w_ is clear, and, in the meantime, accurate SSDEs are required.

The primary aim of this study was to determine the correlations between body weight, BMI, and *d*_w_. Because age is an easily obtainable factor, the correlation between age and *d*_w_ was also assessed to determine whether age can be used as a *d*_w_ surrogate to estimate SSDEs. Unfortunately, unlike the observation that *d*_w_ increases with age for the pediatric population [28], no significant correlation was observed between *d*_w_ and age for chest and abdomen-pelvis CT, which is attributed to the lack of growth of *d*_w_ with the increasing ages of adult patients. However, similar to previous results reported by Menke [20, 25], we also found that both body weight and BMI strongly correlated with *d*_w_ in chest and abdomen-pelvis, despite different populations in Asian-Pacific and European-American regions. Thus, age cannot be used to generate *f* for calculating SSDEs in adult patients. In contrast, both body weight and BMI can be used as *d*_w_ surrogates for accurate patient size, while accounting for geometric dimensions and X-ray attenuation characteristics. However, it is important to note that an automated software program is required to calculate *d*_w_, which must be verified first. Only then is the implementation of *d*_w_ surrogates recommended in clinical practice.

In terms of correlation coefficients, a significant difference was found between body weight and BMI with a common variable of *d*_w_ in chest. However, *d*_w,weight_ was in good agreement with the measured *d*_w_, comparable to *d*_w,BMI_ for chest. Our results also reveal that accurate measurements of patient size, i.e., *d*_w_, can be predicted using both body weight and BMI in abdomen-pelvis. With regard to the accuracy of SSDEs based on body weight and BMI, overall mean root-square-mean errors were less than 6.10% in chest and abdomen-pelvis CTs across all verified patients, although SSDE_weight_ and SSDE_BMI_ had slightly greater dispersion than the reference SSDEs. Additionally, a higher correlation coefficient between BMI and *d*_w_ generated a smaller overall mean root-square-mean error of SSDE_BMI_ compared with SSDE_weight_ in chest and abdomen-pelvis across all verified patients, even when the two values of SSDE_weight_ and SSDE_BMI_ were similar. This result may be attributed to the fact that BMI, which takes height into account, is a better indicator of body size. For males, SSDE_weight_ had a smaller overall mean root-square-mean error than SSDE_BMI_ in the chest CT, while the opposite was true for the abdomen-pelvis region. For females, SSDE_BMI_ had a smaller overall mean root-square-mean error than SSDE_weight_ in either chest or abdomen-pelvis regions. These different results of males and female may be related to the distribution of fat. In general, fat is more distributed in the abdomen-pelvic region, and females tend to have relatively more fat content compared with males [29]. Furthermore, the phenomena can be characterized by BMI to some extent [29]. Hence, our results demonstrate that body weight surrogates were preferable for males in the chest region, and BMI for males in the abdomen-pelvic region and for females in chest and abdomen-pelvic regions, although differences between SSDE_weight_ and SSDE_BMI_ were relatively small. In this study, we further investigated the accuracy of SSDE_weight_ and SSDE_BMI_ for eight different patient BMI populations. Our results indicate that the maximal mean root-square-mean errors were achieved for obese patients, which may be related to the smaller sample size of obese patients in this study.

This study had some limitations. First, it was a retrospective study, and patient choice was a possible bias. However, the patient population was retrieved consecutively. It is likely that the values of *d*_w_ and SSDE obtained were approximated, compared with a prospective study. Second, although the patient population had a wide BMI spectrum, a relatively small sample size of underweight and obese patients was available and no pediatric patients were enrolled. Therefore, further study is needed to determine whether our findings are applicable for overly small and overly large patients, and our results apply only for adult patients. Third, patient data in this study were acquired only from one health institution. As different CT scanners and subtly different CT protocol details, including pitch factor, dose modulation mode, scan range, and respiratory phase, are available in individual health institutions, our results need to be confirmed using data from multiple health institutions. Finally, the BMI of our patients was lower than that of Westerners, although some overweight and obese patients were enrolled in the study. Our results presented correspond to Asian-Pacific body types and may not be generalized for other body types.

## 5. Conclusion

Body weight and BMI were found to closely correlate with *d*_w_ and can be used as *d*_w_ surrogates of anatomic regions of the chest and abdomen-pelvis. On the basis of the linear equations of body weight, BMI, and *d*_w_, SSDEs can be calculated efficiently and accurately in chest and abdomen-pelvis CTs, and the predicted values of SSDE_weight_ and SSDE_BMI_ are comparable to the reference SSDEs derived from axial images with an overall mean root-mean-square error of less than 0.76 mGy or 6.10% for chest and abdomen-pelvis CT. Therefore, we conclude that body weight and BMI can be used to simply and accurately estimate SSDE in the absence of software that automatically calculates SSDEs.

## Figures and Tables

**Table 1:** Demographic data of 1178 patients.

Anatomic region	CT scanner	Number (*n*)	Age (years)	Height (cm)	Body weight (kg)	BMI (kg/m^2^)
Chest	40- and 128- slice	616	58.62 ± 13.47	165.69 ± 8.39	63.52 ± 11.94	23.05 ± 3.46
	40-slice	264	57.33 ± 14.03	165.61 ± 8.47	63.83 ± 12.18	23.15 ± 3.36
	128-slice	352	57.84 ± 13.06	165.87 ± 7.90	63.29 ± 11.77	22.91 ± 3.35

Abdomen-pelvis	40- and 128-slice	562	59.46 ± 12.82	164.74 ± 8.06	62.51 ± 11.50	22.97 ± 3.49
	40-slice	239	58.79 ± 13.72	164.36 ± 7.89	62.39 ± 11.45	23.03 ± 3.47
	128-slice	323	59.93 ± 12.14	165.01 ± 8.19	62.59 ± 11.56	22.93 ± 3.51

Abbreviation: BMI: body mass index.

**Table 2:** Body size and radiation dose of 1178 patients.

Anatomic region	CT scanner	Number (*n*)	*d*_*w*_ (cm)	*f*	CTDI_vol_ (mGy)	SSDE (mGy)
Chest	40- and 128-slice	616	24.47 ± 2.36	1.53 ± 0.16	7.26 ± 2.33	10.66 ± 2.75
	40-slice	264	24.53 ± 2.36	1.53 ± 0.16	6.25 ± 1.81	9.08 ± 1.82
	128-slice	352	24.42 ± 2.37	1.53 ± 0.16	8.02 ± 2.38	11.84 ± 2.73

Abdomen-pelvis	40- and 128-slice	562	25.50 ± 2.37	1.46 ± 0.15	9.66 ± 2.21	13.72 ± 1.83
	40-slice	239	25.38 ± 2.35	1.47 ± 0.15	9.60 ± 2.35	13.65 ± 2.06
	128-slice	323	25.61 ± 2.38	1.46 ± 0.15	9.71 ± 2.12	13.77 ± 1.65

Abbreviation: *d*_w_: water-equivalent diameter; *f*: size-dependent conversion factor; CTDI_vol_: volume CT dose index; SSDE: size-specific dose estimate.

**Table 3:** Mean values and variation of radiation dose in chest and abdomen-pelvic CT examination across all verified patients.

Parameters	Chest			Abdomen		
	SSDE	SSDE_weight_	SSDE_BMI_	SSDE	SSDE_weight_	SSDE_BMI_
Mean values (mGy)	10.15 ± 2.82	10.41 ± 3.06	10.42 ± 2.98	13.54 ± 1.65	13.81 ± 1.98	13.79 ± 1.95
Range (mGy)	13.77	16.83	14.41	10.62	10.70	12.51
COV (%)	27.78	29.39	28.60	12.19	14.34	14.14

Abbreviation: SSDE: size-specific dose estimate; SSDE_weight_: size-specific dose estimate based on body weight; SSDE_BMI_: size-specific dose estimate based on body mass index; COV (%): the coefficient of variation calculated using standard deviation divided by mean values. Note: mean values are reported as the mean ± standard deviation. Range is the difference between maximum and minimum values of radiation dose.

**Table 4:** Mean root-mean-square errors of SSDE_weight_ and SSDE_BMI_ across all verified patients (mGy, %).

Radiation dose	All patients	Males	Females	Underweight	Normal weight	Overweight	Obese
Chest							
Numbers	308	213	95	38	135	127	8
SSDE_weight_	0.62 (6.10%)	0.53 (5.19%)	0.77 (7.69%)	0.41 (5.05%)	0.48 (4.99%)	0.71 (6.38%)	1.49 (10.86%)
SSDE_BMI_	0.57 (5.65%)	0.62 (6.07%)	0.46 (4.60%)	0.40 (4.93%)	0.60 (6.24%)	0.57 (5.13%)	0.84 (5.25%)

Abdomen-pelvis							
Numbers	281	168	113	32	124	120	5
SSDE_weight_	0.76 (5.61%)	0.62 (4.55%)	0.89 (6.64%)	0.61 (5.15%)	0.71 (5.57%)	0.83 (5.67%)	1.14 (6.63%)
SSDE_BMI_	0.71 (5.22%)	0.55 (4.02%)	0.67 (5.00%)	0.73 (6.23%)	0.65 (5.10%)	0.73 (4.98%)	1.23 (7.15%)

Abbreviation: SSDE_weight_: size-specific dose estimate based on body weight; SSDE_BMI_: size-specific dose estimate based on body mass index.

## Data Availability

The data used within the present study are available from the first author.
